# Antigenotoxic Properties of* Agaricus blazei* against Hydrogen Peroxide in Human Peripheral Blood Cells

**DOI:** 10.1155/2017/8759764

**Published:** 2017-02-21

**Authors:** Lada Živković, Sunčica Borozan, Andrea Čabarkapa, Dijana Topalović, Ummi Ciptasari, Vladan Bajić, Biljana Spremo-Potparević

**Affiliations:** ^1^Department of Physiology, Faculty of Pharmacy, University of Belgrade, Belgrade, Serbia; ^2^Department of Chemistry, Faculty of Veterinary Medicine, University of Belgrade, Belgrade, Serbia; ^3^Faculty of Pharmacy, Universitas Gadjah Mada, Yogyakarta, Indonesia; ^4^Laboratory for Radiobiology and Molecular Genetics, Institute for Nuclear Research “Vinca”, University of Belgrade, Belgrade, Serbia

## Abstract

The ability of* Agaricus blazei *mushroom in its dried and powdered mycelial form was evaluated for its antigenotoxic properties for the first time. Antigenotoxic effects in human peripheral blood cells against H_2_O_2_-induced DNA damage were examined in pretreatment and posttreatment protocol by comet assay. The results showed better antigenotoxic properties of* Agaricus blazei *on the interventional level, respectively, after treatment.* Agaricus blazei *in concentration of 250 *μ*g/mL after treatment was most efficient in regard to its action against DNA damage. The evaluation of repair kinetics showed decrease in H_2_O_2_ induced DNA damage 15 min after the application of* A. blazei*, reaching the maximum potency after 30 min. Analysis of antioxidant properties of* Agaricus blazei* revealed strong ^•^OH scavenging properties and moderate reducing power, while its DPPH scavenging ability was weak. In regard to our findings, we can conclude that our preliminary results demonstrated antigenotoxic properties of* Agaricus blazei* and its strong ^•^OH scavenging ability. Mechanisms underlying its properties should be further evaluated in in vivo studies.

## 1. Introduction

*Agaricus blazei*, also known as God's mushroom, Mushroom of life, Royal Sun Agaricus, Mushroom of the Sun, Almond mushroom, or Princess, is coming from southern Brazil [1, 2]. This is an edible mushroom with identified beneficial health effects, like prevention of diabetes, hyperlipidemia, arteriosclerosis, and chronic hepatitis [2].* Agaricus blazei* is vastly used in traditional medicine in the form of a medicinal extract for the prevention and treatment of cancer, as it shows strong immunomodulating properties [3, 4]. Potential antigenotoxic and antimutagen activities have been reported. However, the protective effects of* Agaricus blazei* against well known mutagens have not been established [5–14].

In general, the major composition of the mushrooms is water (90%), protein (2–40%), carbohydrate (1–55%), fiber (3–32%), and ash (8–10%) (ash is mainly composed of salts and metals like calcium and magnesium) [3]. Among the carbohydrates are some biologically active polysaccharides, such as *β*-glucans, that attract the attention of researchers [15]. The polysaccharides phytocomplex is thought to be responsible for its pharmacotoxicological effects [16]. The results of Kozarski et al. indicated that polysaccharide extracts of medicinal mushrooms* Agaricus bisporus* and* Agaricus brasiliensis* act as natural antioxidants [17].

The plentiful presence of free radicals in the environment is associated with the appearance of oxidative stress, which is a basis of aging and the initiation and progress of various diseases and disorders [18]. Hydrogen peroxide causes oxidative DNA damage through the production of a hydroxyl radical (^•^OH-), which can generate multiple DNA modifications, such as base damage, sugar damage, and DNA protein crosslinks. These modifications can ultimately lead to single-strand and double-strands break, inducing genotoxic effects. These alterations can affect the immune response not only in inflammatory diseases but also in cancers [19, 20].

The comet test is a well-established and highly sensitive method that has been used for examining DNA damage and can be applied to assess the genotoxic and genoprotective potentials of several natural products [19–21]. At the same time, the comet assay can be used to measure DNA repair potential in individual cells [19].

A genoprotective activity of the mushroom can play a significant role in prevention and treatment of several mentioned disorders, but very few studies have been done to examine it as a possible therapeutic approach [22, 23]. Menoli et al. observed antigenotoxic potential against DNA damage induced by methyl methanesulfonate in the comet assay [8].

Based on the above findings, the aim of this study was to evaluate genotoxic potential of* Agaricus blazei* on human peripheral white blood cells in vitro in a range of concentrations, to determine its safe nongenotoxic concentrations and to further test its antigenotoxic effects against H_2_O_2_ induced DNA damage. Also, the objective was to assess antioxidant potential of* Agaricus blazei* using reducing power, OH scavenging, and DPPH assays.

## 2. Material and Methods 

### 2.1. Subjects

Peripheral blood samples were collected from three healthy participants: females, between the ages of 20 and 35 years. They did not use cigarettes, alcohols, medicaments, or food supplements in a period of two months. Participants gave their consent in accordance with the regulations of the ethical standards of Ethics Committee for Clinical Trials of the Faculty of Pharmacy, University of Belgrade.

### 2.2. Study Design

In the current study a commercial product of* Agaricus blazei* was tested in the form of capsules (Agaricus blazei, Aloha Medicinals, Inc, Carson City, NV, USA/Santa Cruz, CA, USA facility). The capsules contained the mushroom in its mycelial form which had been grown on white sorghum, aged, dried, and powdered. The powder was dissolved in phosphate-buffered saline (PBS), stirred for 30 min at 37°C, and filtered through a filter paper. Three concentrations of 250, 500, and 1000 *μ*g/mL were made to perform the comet assay experiments.

#### 2.2.1. Genotoxic and Antigenotoxic Assessment of* Agaricus blazei* by Comet Assay

A comet assay was performed to evaluate the genotoxic and antigenotoxic properties of* Agaricus blazei *mushroom. A concentration of 50 *μ*M H_2_O_2_ was chosen to induce a consistent level of DNA damage in human peripheral white blood cells. Suspension of human peripheral blood cells was embedded in a 0.67% low-melting-point agarose and spread to microscopic slides. The cells were exposed to the following treatments: (1) to evaluate the genotoxicity of the mushroom, the cells were exposed to* Agaricus blazei* in three different concentrations (250, 500, and 1000 *μ*g/mL) for 30 min at 37°C; two protocols were used to evaluate antigenotoxic potential, before treatment and after treatment: (2) before treatment, the cells were incubated with the* Agaricus blazei* prior to their exposure to H_2_O_2_ (an assessment of the mushrooms' action at the preventive level); and (3) after treatment, the cells were treated with the mushroom after their exposure to H_2_O_2_ (an assessment of the mushrooms' action at the interventional level). The cells were treated with* A. blazei* for 30 min at 37°C, while treatment with H_2_O_2_ was conducted for 20 min at 4°C.

In the antigenotoxic assessment, a solution of a well known antioxidant, quercetin in PBS, was used as a positive control. Quercetin has demonstrated high antioxidant and antigenotoxic activity previously in our experimental conditions for tested concentrations: 100, 250, and 500 *μ*g/mL, whereas H_2_O_2_ treatment of the cells was performed as a negative control.

For the test of genotoxicity, the cells were treated with PBS as a negative control and H_2_O_2_ as a positive control.

#### 2.2.2. Kinetics of Attenuation of H_2_O_2_-Induced DNA Damage with* Agaricus blazei* after Treatment by Comet Assay

To evaluate the repair kinetics in H_2_O_2_-induced DNA damage after treatment with* Agaricus blazei*, the most effective concentration was chosen from previous posttreatment tests. Following 20 min of H_2_O_2_ (50 *μ*M) treatment at 4°C, cells that have been treated with the* A. blazei *were incubated at 37°C for 4 time periods: 15, 30, 45, and 60 min.. Simultaneously, for the positive controls, the cells were treated with PBS and examined at the same intervals.

### 2.3. The Single Cell Gel Electrophoresis Assay (Comet Assay)

The viability of cells used in different treatments was checked with trypan blue exclusion method. For the estimation of the dead cell fraction, cell samples were stained with a 0.4% solution of trypan blue in PBS. The number of blue-stained (dead) cells within 2,000 cells was counted on haemocytometer. The cell viability was above 90%.

The comet assay was performed essentially as described by Singh et al. [24]. Briefly, 6 *μ*L of whole blood samples was suspended in 0,67% low-melting-point agarose (LMP) (Sigma-Aldrich, St. Louis, MO) and pipetted onto superfrosted glass microscope slides precoated with a layer of 1% of normal-melting-point agarose (Sigma-Aldrich, St. Louis, MO), spread by a coverslip, and maintained for 5 min in the freezer to solidify. After gently removing the coverslips, the cell suspensions on slides were treated with tested mushroom and H_2_O_2_ as described above in three mentioned types of experiments. Following the treatments, all slides were covered with the third layer of 0,5% LMP agarose and again allowed to solidify in the freezer for 5 min. After removal of the coverslips, the slides were placed in cold lysing solution (2,5 M NaCl, 100 mM EDTA, 10 mM Tris, 1% Triton X 100 and 10% dimethylsulfoxide, pH 10 adjusted with NaOH) at 4°C overnight. The next day, the slides were removed from lysing solution, placed in the horizontal gel electrophoresis tank (CHU2 manufacturer, connected to a Power Supplier EPS 601), and flooded with cold fresh electrophoresis buffer (10 M NaOH, 200 mM EDTA) allowing the DNA to denature for 30 min before electrophoresis. The electrophoresis was conducted in dimmed light at 25 V and 300 mA for 30 min, and slides were afterwards stained with ethidium bromide, performed as described by Singh et al. [24]. The comets were observed and analyzed using Olympus 50 microscope (Olympus Optical Co., GmbH Hamburg, Germany), equipped with a fluorescence recording device at 100x magnification.

**Figure 1:**
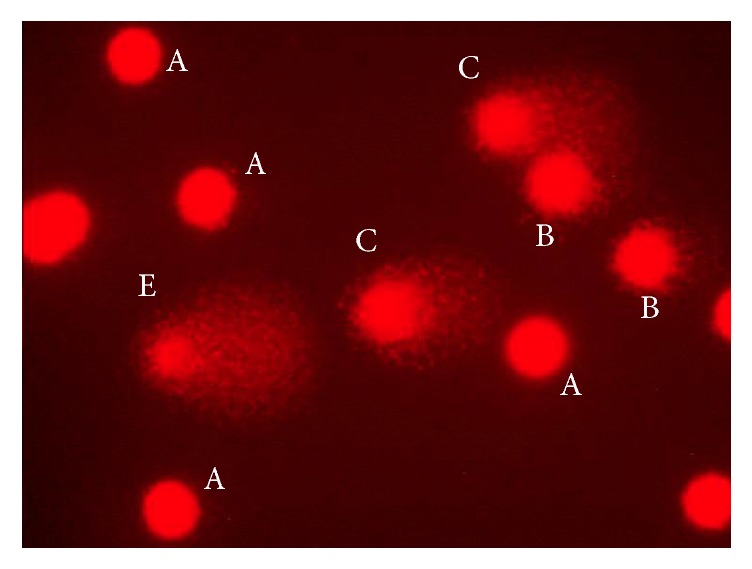
The example of comet assay slide preparation with different classes of comets (cell nuclei stained with ethidium bromide).

The numbers of comets were used as parameter that reflected DNA damage. Nuclei of cells, resembling comets, were graded by eye inspection into 5 classes, depending on the extent of DNA damage, as described by Anderson et al. [19] representing (1) class A: undamaged cells with no comet tail (<5% damaged DNA); (2) class B: low-level damage (5%–20%); (3) class C: medium-level damage (20%–40%); (4) class D: high level damage (40%–95%); and (5) class E: total destruction (>95%). The example of comet assay slide preparation with different classes of comets is given in Figure 1. DNA damage was characterized as DNA migration when the damage was more than 5% (B + C + D + E comet classes). The mean value was calculated for 100 comets total per subject (the mean number from 100 on each of two slides), for each experiment. Apoptotic and necrotic cells were excluded from the analyses.

### 2.4. Reducing Power Assay

The reducing power was determined according to the method of Oyaizu [25]. Each concentration of* A. blazei *(0,062–2 mg/mL) in 50% DMSO deionized water was mixed with 2,5 mL of 200 mM/L sodium phosphate buffer (pH 6,6) and 2,5 mL of 10 mg/mL potassium ferricyanide, and the mixture was incubated at 50°C for 20 min. Afterwards 2,5 mL of 100 mg/mL trichloroacetic acid was added to the mixture and then centrifuged at 200*g* for 10 min. The upper layer was mixed with 5 mL of deionized water and 1 mL of 1 mg/mL ferric chloride; the absorbance was then measured at 700 nm against a blank. A higher absorbance value indicated a higher reducing power. Butylated hydroxytoluene (BHT) was used as standard. The concentrations of the* Agaricus blazei* and BHT used for the analysis were in a range of 0.019, 0.039, 0.078, 0.156, 0.132, 0.625, 1.25, 2.50, and 5.00 mg/mL.

### 2.5. Determination of DPPH Radicals Scavenging Ability

The scavenging ability on 1,1-diphenyl-2-picrylhydrazyl (DPPH) radicals was determined according to the method of Shimada et al. [26]. Each concentration of* A. blazei* (0,062–2 mg/mL) in 50% DMSO was mixed with 1 mL of methanolic solution containing DPPH (Sigma-Aldrich, St. Louis, MO) radicals, resulting in a final concentration of 0,2 mM/L DPPH. The mixture was shaken vigorously and left to stand for 30 min in the dark. The absorbance was then measured at 517 nm against a blank. The scavenging ability was calculated as follows:

(1) Scavenging  ability%=ΔA517  of  control−ΔA517  of  sampleΔA517  of  control×100.

IC_50_ value (mg/mL) shows the effective concentration at which 50% of DPPH radicals were inhibited and were obtained by interpolation from linear regression analysis. Trolox was used as standard for comparison. The concentrations of the* Agaricus blazei* and Trolox used for the analysis were in a range of 0.062, 0.125, 0.250, 0.500, 1.00, and 2.00 mg/mL.

### 2.6. Determination of Hydroxyl Radical Scavenging Activity

The effect of hydroxyl radical was assayed using the 2-deoxyribose oxidation method described in Chung et al. [27]. 2-Deoxyribose is oxidized by the hydroxyl radical that is formed by the Fenton reaction and degraded to malondialdehyde. The reaction mixture contained 0,45 mL of 0,2 M sodium phosphate (pH 7,6), 0,15 mL of 10 mM 2-deoxyribose, 0,15 mL of 10 mM FeSO4-EDTA, 0,15 mL of 10 mM hydrogen peroxide, 0,525 mL of distilled water, and 0,075 mL (0,062–2 mg/mL) of* A. blazei* solution in a tube. The reaction was started by the addition of hydrogen peroxide. After incubation at 37°C for 1 h, the reaction was stopped by adding 0,75 mL of 2,8% (w/v) trichloroacetic acid and 0,75 mL of 1.0% (w/v) of thiobarbituric acid. The mixture was boiled for 10 min., cooled in ice, and then measured at 535 nm. The reaction mixture not containing test sample was used as the control. Trolox (0,0078–2 mg/mL) was used as standard antioxidant. The scavenging activity on hydroxyl radicals was expressed as follows:

(2) Scavenging  ability%=ΔA535  of  control−ΔA520  of  sampleΔA535  of  control×100.

IC_50_ value (mg/mL) was the effective concentration by which 50% of 2-deoxyribose was degraded and was obtained by interpolation from linear regression analysis. Trolox was used as standard for comparison. The concentrations of the* Agaricus blazei* and Trolox used for the analysis were in a range of 0.007, 0.015, 0.031, 0.062, 0.125, 0.250, 0.500, 1.00, and 2.00 mg/mL.

### 2.7. Statistical Analysis

The statistical analysis was performed using a Kruskal Wallis test, with Dunn's post hoc for comparisons of different treatments versus the respective controls. Data were expressed as mean ± standard error of the mean (SEM), with *n* = 3. A difference at *p* < 0,05 was considered statistically significant. GraphPad Prism (5.0) statistical software (GraphPad Software Inc, La Jolla, CA, USA) was used for the analysis.

## 3. Results

### 3.1. Genotoxic Properties of* Agaricus blazei*

**Figure 2:**
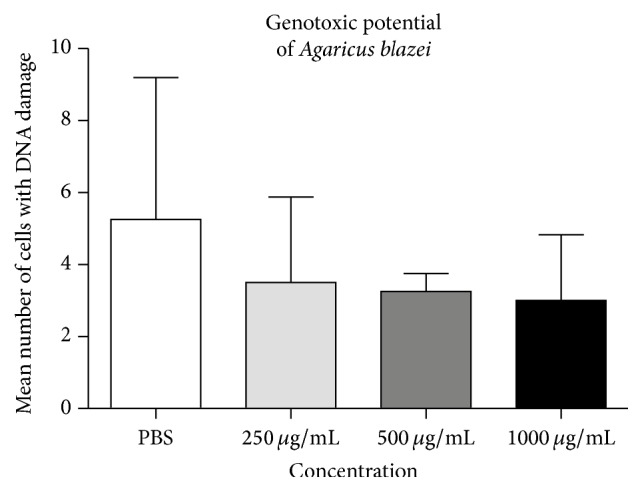
The evaluation of genotoxic effects of* Agaricus blazei* in 3 tested concentrations after 30 min of incubation at 37°C. Bars represent mean number of cells with DNA damage ± SEM, for *n* = 3.

In the assessment of the genotoxic potential of* Agaricus blazei*, it was found that the tested range of concentrations applied to agarose-embedded blood cells did not increase DNA migration in comparison to the negative control (i.e., the PBS) (Figure 2). Moreover, for all tested concentrations, the basal level of primary lesions decreased in regard to the control. Based on our results, the* Agaricus blazei* in the tested concentrations indicated a lack of a genotoxic effect.

### 3.2. Antigenotoxic Properties of* Agaricus blazei*

The antigenotoxic effects of* Agaricus blazei* were examined under two experiment protocols: pretreatment with the mushroom prior to H_2_O_2_ exposure and posttreatment with the mushroom after the treatment with H_2_O_2_ that causes oxidative DNA damage through the production of a hydroxyl radical (^•^OH).

**Figure 3:**
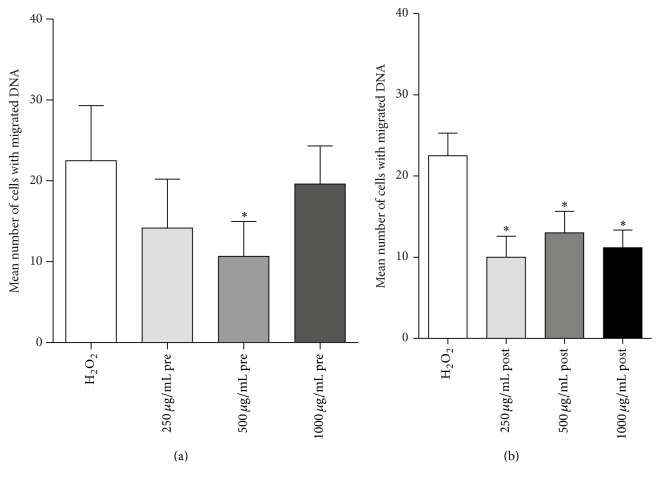
The evaluation of antigenotoxic properties of* Agaricus blazei* against DNA damage induced by H_2_O_2_ in (a) pretreatment and (b) posttreatment protocol. Bars represent mean number of cells with DNA damage ± SEM, for *n* = 3; ^*∗*^*p* < 0.05, versus H_2_O_2_ treated control (by Kruskal Wallis and Dunn's post hoc test).

Figure 3(a) shows that pretreatment application of the mushroom displayed slight reduction in the mean number of damaged cells, where concentration of 500 *μ*g/mL exhibited significant attenuation in comparison to the control (cells treated with 50 *μ*M H_2_O_2_). Interestingly, the U-shaped concentration-dependent trend could be noticed in the pretreatment attenuation of the DNA damage induced by H_2_O_2_.

Posttreatment conditions are displayed in Figure 3(b). Results showed that all three concentrations significantly decreased the number of cells with DNA damage (*p* < 0,05), displaying an antigenotoxic effect of* Agaricus blazei *at an intervention level.

**Figure 4:**
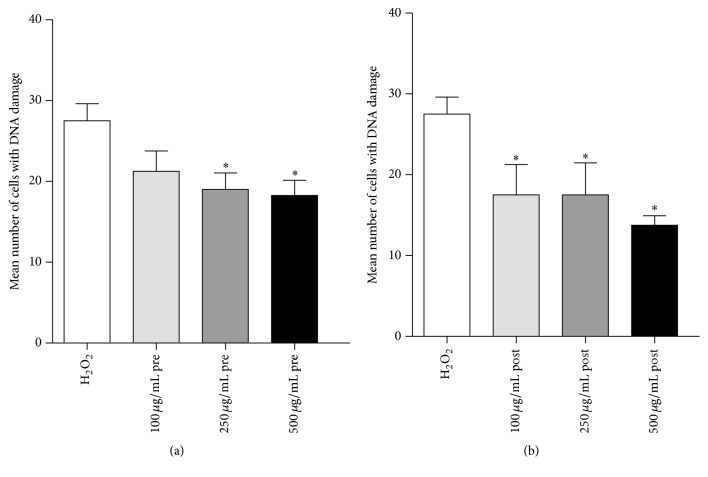
The evaluation of antigenotoxic properties of quercetin against the DNA damage induced by H_2_O_2_ in (a) pretreatment and (b) posttreatment protocol. Bars represent mean number of cells with DNA damage ± SEM, for *n* = 3; ^*∗*^*p* < 0.05, versus H_2_O_2_ treated control (by Kruskal Wallis and Dunn's post hoc test).

Figure 4 shows the results of positive controls in which the cells were treated with 100, 250, and 500 *μ*g/mL of quercetin, where the percentage of DNA damaged cells decreased in a concentration-dependent manner for both pretreatment and posttreatment experiment. However, the antigenotoxic effect of quercetin was more profound in posttreatment experiment, as well.

**Figure 5:**
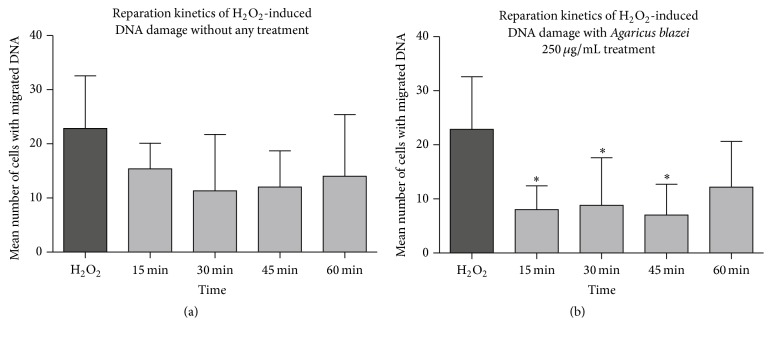
A Time course of DNA damage repair of cells exposed to H_2_O_2_ and afterwards incubated for 15, 30, 45, and 60 min (a) without any treatment; (b) with 250 *μ*g/mL of the* Agaricus blazei*. Bars represent mean number of cells with DNA damage ± SEM, for *n* = 3; ^*∗*^*p* < 0.05, versus H_2_O_2_ as control (by Kruskal Wallis and Dunn's post hoc test).

According to posttreatment results of* Agaricus blazei*, the concentration of 250 *μ*g/mL was chosen as the most efficient in action on intervention level. It was later used to investigate a time course of DNA repair (the reparation kinetics in H_2_O_2_ induced DNA damage). The results of attenuation of H_2_O_2_-induced DNA damage in 4 times periods: 15, 30, 45, and 60 min, both in cells treated with* A. blazei* and in control, are presented in Figure 5. In the control, where cells were incubated for the mentioned periods without any additional treatment after the H_2_O_2_ exposure, the levels of H_2_O_2_-induced DNA lesions were not significantly decreased (Figure 5(a)), although data showed that untreated cells displayed potential to attenuate DNA damage. However, it should be noted that, in the presence of* A. blazei* at the time points 15, 30, and 45 min, there was significant attenuation of H_2_O_2_ induced damage (Figure 5(b)).

### 3.3. Determination of Reducing Power of* Agaricus blazei*

**Figure 6:**
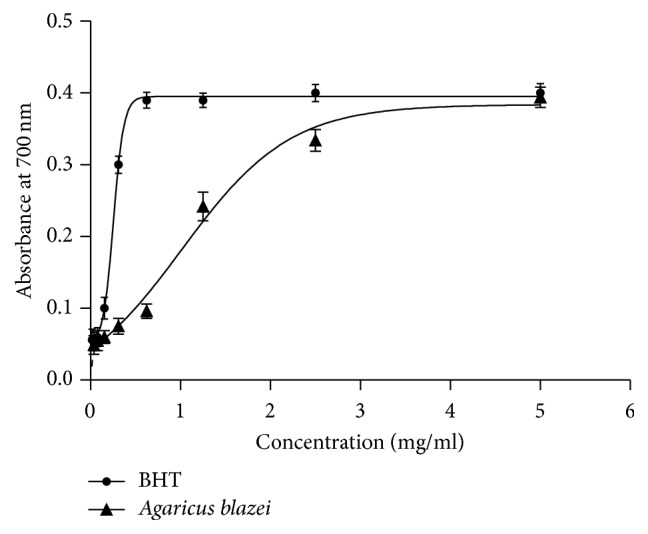
The reducing power of* Agaricus blazei* compared to BHT.

The reducing power of* A. blazei *is presented in Figure 6. In tested concentrations (0,062–5 mg/mL)* A. blazei *showed concentration-dependent moderate reducing power ability when compared to BHT as control.

### 3.4. Determination of DPPH Radical Scavenging Activity of* Agaricus blazei*

The DPPH^•^ radical scavenging effects of* A. blazei* are presented in Table 1. Results show that* A. blazei* in concentrations used in this test has no free radical scavenging activity. The free radical scavenging activity of* A. blazei* was compared to Trolox, a synthetic antioxidant.* A. blazei* of 2 mg/mL showed only 3% inhibition of DPPH.

### 3.5. Determination of Hydroxyl Radical Scavenging Activity of* Agaricus blazei*

**Figure 7:**
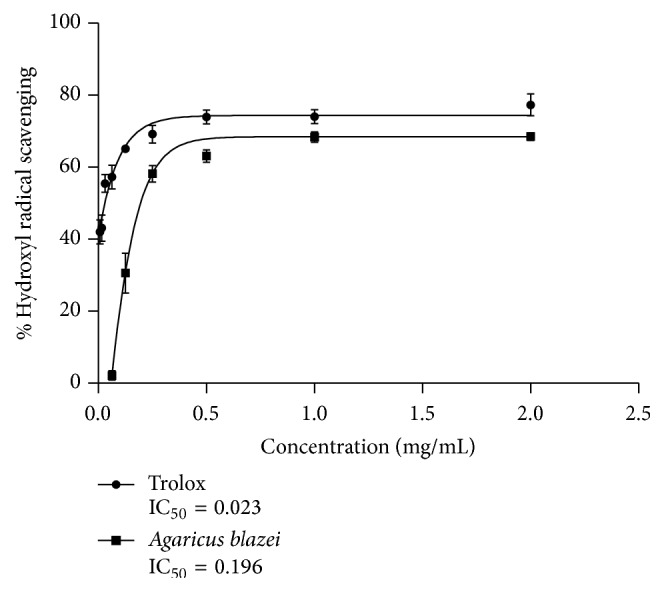
Hydroxyl radical scavenging activity of* Agaricus blazei* in a range of concentrations (0,007–2 mg/mL) compared to Trolox.

Figure 7 shows the hydroxyl radical scavenging effects determined by the 2-deoxyribose oxidation method. The scavenging effect of* A. blazei* on hydroxyl radical showed 50% inhibition for all tested concentrations above 0,196 mg/mL (IC_50_ = 0,196 mg/mL), while Trolox as standard at the same concentration showed 60% inhibition. These results indicate an excellent hydroxyl radical scavenging activity of* Agaricus blazei*.

## 4. Discussion

In this work, we assessed the genotoxicity and antigenotoxicity effects of* A. blazei* on human peripheral blood cells. DNA strand breaks were measured using a simple and reproducible technique, the comet assay. Compared to many other macromolecules, DNA is a sensitive molecule and damage may result from exposure to exogenous agents. Prolonged or repeated DNA damage and genomic instability can contribute to multiple diseases including cancer [28].

In the current study, no genotoxic effects of* A. blazei* on human peripheral blood cells were determined. This is in agreement with the reports by Radaković et al. [29] and Guterrez et al. [10, 12]. Namely, the exposure of cells to the mushroom in our experiment even leads to a reduction of the baseline level of DNA damage. Meanwhile, the beneficial role of* Agaricus blazei* in reduction of H_2_O_2_ induced DNA damage in whole blood cells was demonstrated. As a positive control quercetin was used. Quercetin is a standard antioxidant that effectively reduces H_2_O_2_-induced DNA damage [30]. The reduction of DNA damage by quercetin in this experiment was a confirmation that a ROS attack on the DNA underlies the origin of strands breaks. Therefore, it can be noted that* Agaricus blazei* also exhibited effective attenuation of free radical induced DNA strand breaks.

To elucidate the possible mechanism behind the antigenotoxic action of the* A. blazei*, two approaches were applied in order to investigate whether the mushroom could act at the preventive and the interventional level. In pretreatment conditions,* A. blazei* was added to cells 30 min before administration of the oxidant, allowing them to be active at the prevention level [31]. Under pretreatment conditions the mushroom may act by increasing the antioxidant capacity of the cells, making them more resistant to oxidative DNA damage [32]. The results of pretreatment showed moderate ability to decrease the number of DNA damaged cells, where 500 *μ*g/mL concentration displayed a significant protective effect against H_2_O_2_. It was shown by de Sá-Nakanishi et al. [33] that the administration of* A. blazei* was protective to the brain of old rats against oxidative stress by increasing the brains enzymatic antioxidant capacity, showing an inducible effect on their activities. Also, the action of an* A. blazei* pretreatment on the paracetamol injury in rats exhibited the ability of boosting the levels of the catalase [34].

On the other hand, posttreatment data displayed a significant decrease in the number of cells with DNA damage at all tested concentrations showing prominent interventional activities of* A. blazei*. Antigenotoxic effect of* A. blazei* seen after treatment could be assigned to synergistic action of independent mechanisms which possibly contributed in DNA damage reduction: free radical scavenging and stimulation of DNA repair.

It has been established that mushrooms can demonstrate their antioxidant properties at different stages of the oxidation process and by different mechanisms [35]. The antioxidant properties in this study were examined by the reducing power method and scavenging abilities on OH radicals and DPPH radicals. The reducing power of* Agaricus blazei* showed a concentration-dependent trend and was 0,3 at 2 mg/mL which is considered as a moderate power. This is in accordance with previous results shown by Tsai et al. [36] that demonstrated similar reducing power of ethanolic extract of* A. blazei*.

The scavenging ability is important antioxidant protection against high reactivity of free radicals. Our data on DPPH scavenging ability showed that the activity of* A. blazei* was extremely modest. This could be explained by the fact that the antioxidant activity of natural compounds is associated with their structures, and their accessibility to the radical centre of DPPH^•^ could influence their antioxidant power [37]. It should be emphasized that we evaluated the ability of* Agaricus blazei* as a mushroom in its dried and powdered mycelial form dissolved in PBS, so the structure of its compounds could affect its antioxidant abilities. Work by Tsai et al. [36] showed different antioxidant properties of* A. blazei* dependent on the extraction procedure. On the other hand,* A. blazei *in our study showed pronounced OH scavenging properties for the concentrations above 0,196 mg/mL. Hydroxyl radical is the most reactive chemical species known to induce oxidative damage to proteins, lipids, and DNA. Accordingly, the compounds with OH scavenging properties are of extreme importance in biological systems. It should be noted that the application of* A. blazei *in the posttreatment comet assay provided the best attenuation of DNA damage at the 250 *μ*g/mL concentration and which at the same time showed 70% inhibition of OH radical.

In order to assess the next mechanism that could have contributed to the posttreatment efficiency, a time course of DNA repair in the cells treated with the optimal concentration of* Agaricus blazei *was investigated and compared with untreated cells (exposed only to PBS). Data showed that posttreatment with the mushroom significantly decreased the level of cells with DNA damage in the 15, 30, and 45 time course, while the cells treated only with PBS did not display significant reduction of the DNA damage in the same time frame. It should be mentioned that a similar trend in DNA damage reduction was detected in both experimental conditions: a decrease in DNA damage was observed already at 15 min, reaching the maximum reduction after 45 min. Previous studies showed that significant repair of DNA damage occurred within 1 h after the exposure to the oxidative agent [38]. Therefore, those results indicate that* Agaricus blazei* could significantly stimulate the repair process in DNA damaged cells, and the repair capacity of cells treated with mushroom is more efficient than the repair capacity of untreated cells. Study of Da Silva et al. [39] showed protective effect of *β*-glucan extracted from* Agaricus blazei* on the expression of the genes ERCC5 (involved in excision repair of DNA damage) on HepG2 cells.

Bearing in mind that efficiency of pretreatment can be explained by* Agaricus blazei *property of being able to increase the cells' antioxidant capacity, possible synergistic (additional) mechanism of posttreatment action could be the antioxidant enzymes stimulation.

Summarizing previous findings,* Agaricus blazei *activity on the interventional level can be attributed to its scavenging properties, stimulation of DNA repair, as well as additional antioxidant enzyme activation. It should be especially emphasized that for the first time it has been revealed that* Agaricus blazei* has strong potential in reduction of OH groups.

Our study was conducted as an in vitro test, which should contribute to highlighting* Agaricus blazei* as an antioxidant that attenuates the impact of oxidative stress to DNA. According to these findings, it is necessary to perform further investigation of its properties on in vivo systems.

## 5. Conclusion

*Agaricus blazei* is under investigation for a broad range of applications. This preliminary study shows antigenotoxic properties of* Agaricus blazei* on human peripheral blood cells against H_2_O_2_-induced DNA damage on the interventional level. Also, for the first time, it was shown that* Agaricus blazei* has strong OH scavenging properties. Mechanisms underlying the antigenotoxic properties should be further evaluated in in vivo studies.

## Figures and Tables

**Table 1:** DPPH scavenging ability of *Agaricus blazei* compared to Trolox, expressed as % inhibition (*n* = 3).

Percentage inhibition %		
Amount (mg/mL)	*Agaricus blazei*	Trolox
0.062	0.33 ± 0.08	18.99 ± 0.29
0.125	0.62 ± 0.12	42.15 ± 0.22
0.25	0.80 ± 0.05	90.12 ± 0.30
0.5	0.99 ± 0.12	96.00 ± 0.04
1	1.70 ± 0.15	95.96 ± 0.08
2	3.16 ± 0.22	96.25 ± 0.21
